# Partial Human Combustion

**Published:** 1835

**Authors:** 


### VIII.—Partial Human Combustion.

*An Essay on Spontaneous Combustion, read before the Medical Society of the State of Tennessee, at their Annual Meeting in May,* 1835. By James Overton, M. D.

The curious phenomenon of human combustion, from, or without, the contact of fire, has already been brought before the readers of this journal. In the second volume, p. 130–141, is a translation from the French, of a comprehensive memoir on this subject, by M. Marc. In looking into the Essay of Dr. Overton, we do not observe, that since the time when M. Marc wrote his paper, there has been any new suggestion as to the proximate cause of this curious event.

An extraordinary accumulation of hydrogen gas in the cellular tissue of the body, set on fire by an electric spark, or a burning body—the theory of M. Marc, is that of Dr. Overton. The Doctor has, however, made a substantial addition to our previous stock of facts, by a minute account of a case of partial spontaneous human combustion, which lately occurred in Tennessee; and it is chiefly to make a permanent record of this case, that we have introduced the subject.

Prof. H., of the University of Nashville, is a gentleman 35 years old, of middle size, light hair, hazle eyes, and sanguino-lymphatic temperament; he has been extremely temperate as to alcoholic stimulation of every kind; led a sedentary and studious life; and been subject to a great variety of dyspeptic affections. On the 5th of January, 1835, he left his recitation room at 11 o'clock, A M., and walked briskly, with his surtout buttoned round him, to his residence, three quarters of a mile. The thermometer was at 8°, and the barometer at 29.248—the sky clear and calm. On reaching home he engaged in meteorological observations, and in 30 minutes, while in the open air about to record the direction of the winds—

"He felt a pain as if produced by the pulling of a hair, on the left leg, and which amounted in degree to a strong sensation. Upon applying his hand to the spot pained, the sensation suddenly increased, till it amounted in intensity to a feeling resembling the continued sting of a wasp or hornet. He then began to slap the part by repeated strokes with the open hand, during which time the pain continued to increase in intensity, so that he was forced to cry out from the severity of his suffering. Directing his eyes at this moment to the suffering part, he distinctly saw a light flame of the extent, at its base, of a ten cent piece of coin, with a surface approaching to convexity, somewhat flattened at the top, and having a complexion which nearest resembles that of pure quicksilver. Of the accuracy in this latter feature in the appearance of the flame, Mr. H. is very confident, notwithstanding the unfavorable circumstances amidst which the observation must have been made. As soon as he perceived the flame, he applied over it both his hands open, united at their edges, and closely impacted upon and around the burning surface. These means were employed by Mr. H. for the purpose of extinguishing the flame by the exclusion of the contact of the atmosphere, which he knew was necessary to the continuance of every combustion. The result was in conformity with the design, for the flame immediately went out. As soon as the flame was extinguished, the pain began to abate in intensity, but still continued, and gave the sensation usually the effect of a slight application of heat or fire to the body, which induced him to sieze his pantaloons with one of his hands and to pinch them up into a conical form over the injured part of the leg, thereby to remove them from any contact with the skin below. This operation was continued for a minute or two, with a design of extinguishing any combustion which might be present in the substance of his apparel, but which was not visible at the time. At the beginning of the accident, the sensation of injury was confined to a spot of small diameter, and in its progress the pain was still restricted to this spot, increasing in intensity and depth to a considerable extent, but without much if any enlargement of the surface which it occupied at the beginning. A warmth was felt to a considerable distance around the spot primarily affected, but the sensation did not by any means amount in degree to the feeling of *pain.* This latter sensation was almost, if not entirely confined to the narrow limits which bounded the seat of the first attack, and this sensation was no otherwise modified during the progress of the accident, than by its increasing intensity and deeper penetration into the muscles of the limb, which at its greatest degree seemed to sink an inch or more into the substance of the leg.

Believing the combustion to have been extinguished by the means just noticed, and the pain having greatly subsided, leaving only the feeling usually the effect of a slight burn, he untied and pulled up his pantaloons and drawers, for the purpose of ascertaining the condition of the part which had been the seat of his suffering. He found a surface on the outer and upper part of the left leg, reaching from the femoral end of the fibula in an oblique direction, towards the upper portion of the grastrochnemi muscles, about three-fourths of an inch in width, and three inches in length, denuded of the scarfskin, and this membrane gathered into a roll at the lower edge of the abraded surface. The injury resembled very exactly in appearance an abrasion of the skin of like extent and depth, often the effect of slight mechanical violence, except that the surface of it was extremely *dry,* and had a complexion more livid than that of wounds of a similar extent produced by the action of mechanical causes." pp. 25-26.

His drawers, composed of silk and wool, immediately over the abraded skin, were burnt entirely through, but the scorching had not extended in the slightest degree beyond. The pantaloons, made of broadcloth, were uninjured; but over the affected spot the extremities of the wool were tinged with a kind of dark, yellowish matter, which could be easily scraped off with a knife.

"Considering the injury not to be of a serious character, Mr. H. bestowed upon its treatment no particular care or attention, but pursued his usual avocations within doors and in the open air, which was very cold, until the evening of the succeeding day. At this time the wound became inflamed and painful, and was dressed with a salve, into the composition of which the rosin of turpentine entered in considerable proportion. This treatment was continued for four or five days, during which time the wound presented the usual aspect of a burn from ordinary causes, except in its greater depth and more tardy progress towards cicatrization, which did not take place till after thirty-two days from the date of the infliction of the injury. The part of the ulcer which healed last was the point of inception and intensity of the pain at the time of attack, and which point was evidently the seat of deeper injury than any other portion of the wounded surface. About the fifth day after the accident, a physician was requested to take charge of the treatment, and the remedies em loyed were such chiefly, as are usual in the treatment of burns from other causes, except that twice a week the surface of the ulcer was sprinkled over with calomel, and a dressing of simple cerate applied above it. In the space between the wound and the groin there was a considerable soreness of the integuments to the touch, which continued during the greatest violence of the effects of the accident, and then gradually subsided. The cicatrix is at this time, March 24th, entire; but the surface is unusually scabrous, and has a much more livid aspect than that of similar scars left after the infliction of burns from common causes. The dermis seemed to have been less perfectly regenerated than is usual from burns produced by ordinary means, and the circulation through the part is manifestly impeded, apparently in consequence of atony of its vessels, to an extent far beyond any thing of a similar nature to be observed after common burns." pp. 27-28.

